# Draft genome sequence of Flavobacterium aquidurense strain, isolated from untreated wastewater

**DOI:** 10.1099/acmi.0.000976.v3

**Published:** 2025-06-20

**Authors:** Alexander D. H. Kingdon, Kara D’Arcy, Anya Breen, Claudia McKeown, Ellie Allman, Priyanka Sharma, Amy McLeman, Adam P. Roberts

**Affiliations:** 1 Department of Tropical Disease Biology, Liverpool School of Tropical Medicine, Liverpool, UK

**Keywords:** antimicrobial resistance, beta-lactamase, *Flavobacterium*, genome sequencing, mobile genetic elements, wastewater

## Abstract

Here, we report the draft 5.8 Mb genome sequence of a *Flavobacterium aquidurense* isolate from untreated wastewater in Liverpool, United Kingdom. The reported isolate has the potential to produce both flexirubin and β-carotene pigments, and contains an additional biosynthetic gene cluster for a putative novel β-lactone. The genome also contains a gene for a putative β-lactamase *bla_JOHN-1_* analogue, and there are multiple copies of a putative novel insertion sequence of the IS*3* family. This genome adds to a growing resource of *Flavobacterium* spp*.* sequencing data which can be utilized to investigate microbial pigment production, antimicrobial resistance genes and mobile genetic elements within this genus.

## Data Availability

The data are available as part of BioProject PRJNA1161700. The raw reads have been deposited in SRA under the accession no. SRR30678054. The assembled and annotated genome has been deposited in GenBank, under accession no. JBIEAU000000000.

## Introduction

*Flavobacterium* spp. are commonly isolated from soil and water samples [1,3], and in recent years from Antarctic habitats [4,6], and are considered opportunistic pathogens in several fish species [7,9]. There have been limited reports of human infections caused by *Flavobacterium* spp., specifically *F. ceti* [10,11] and *F. lindanitolerans* [12,13]. We report here the genome sequence of a *Flavobacterium* spp. isolate from untreated wastewater from Liverpool, United Kingdom.

## Methods

Untreated wastewater was collected from the outflow of the Liverpool Life Sciences Accelerator, part of the Liverpool School of Tropical Medicine (53°24′ N, 2°58′ W, United Kingdom) in March 2021 [14]. Sterile swabs were used to spread wastewater onto brain–heart infusion (BHI) agar followed by incubation at room temperature for 2 days. Individual colonies of bacteria were collected and stored as part of the Swab and Send project [15]. The isolate was cultured statically in BHI broth at room temperature for 2 days, before being stored in 20% glycerol at −70 °C. The organism was preliminarily identified using 16S rRNA primers 27F (AGAGTTTGATCCTGGCTCAG) and 1492R (GGTTACCTTGTTACGACTT) in a colony PCR, followed by Sanger sequencing (Azenta). The amplicon sequence was run through BLASTn against the core nucleotide database [16]. The closest match to this partial sequence was *Flavobacterium frigidimaris* DSM15937, an isolate from Antarctic seawater [5], accession no. NR_041057.1. Due to the large differences in the environment of isolation, we decided to obtain the *Flavobacterium aquidurense* genome sequence.

A single orange colony was cultured in BHI broth, shaking at 250 r.p.m., at 28 °C, until mid-log was reached. Once at an OD_600_ of 0.9, 10 ml of culture was pelleted, transferred to 500 µl DNA/RNA Shield (Zymo Research, USA) and sent to MicrobesNG (https://microbesng.com/) for processing as follows: An aliquot of this cell suspension (40–50 µl) was lysed using tris ethylenediaminetetraacetic acid (TE) buffer containing lysozyme, metapolyzyme and RNase A (120 µl) incubated at 37 °C for 25 min. To this mixture, proteinase K (final concentration=0.1 mg ml^−1^) and SDS (final concentration=0.5% v/v) were added and incubated at 65 °C for 5 min. DNA was purified using solid-phase reversible immobilization beads, then resuspended in elution buffer (EB, equal volume). Library preparation was performed using the Nextera XT Library Prep Kit (Illumina, USA) following the default protocol, except the input DNA was increased two-fold and the PCR elongation time increased to 45 s. An Illumina NovaSeq 6000 was used for short-read sequencing using a 250 bp paired end protocol. This resulted in 1,091,197 raw reads. For all software processing, the default parameters were used unless stated. The read adapters were trimmed using Trimmomatic v.0.30 with a sliding window quality cut-off of Q15 [17]. Genome assembly was undertaken using SPAdes v.3.7 [18] and annotated using NCBI’s Prokaryotic Genome Annotation Pipeline v.6.8 [19].

## Genome description

The reported *Flavobacterium* sp. isolate genome has a total size of 5,799,299 bp and overall GC content of 34.85%. The mean coverage across the genome was 88.26×. Genome assembly resulted in 60 contigs with an N_50_ of 436,906 bp. The genome assembly had a completeness of 99.22% and contamination of 0.89%, as assessed by CheckM [20]. Across these contigs, there were 4,921 predicted genes, plus 63 tRNA sequences. The draft genome was compared to the closest 16S rRNA match, *F. frigidimaris* DSM15937 (MUGV00000000), revealing low genome similarity with an average nucleotide identity (ANI) of 82.23 (44.15% coverage) [21]; however, the closest match was to *Flavobacterium aquidurense* DSM18293 (MUGR00000000), ANI 98.58 (63.37% coverage) [8]. ANI was calculated using EZBioCloud’s webserver (https://www.ezbiocloud.net/tools/ani), which used the OrthoANIu algorithm. Retrospective comparison to the *gyrB* gene also highlighted *F. aquidurense* DSM18293 as the closer match, having an amino acid sequence identity of 100% (100% coverage), compared to 96.6% (100% coverage) against *F. frigidimaris* DSM15937.

**Fig. 1.**
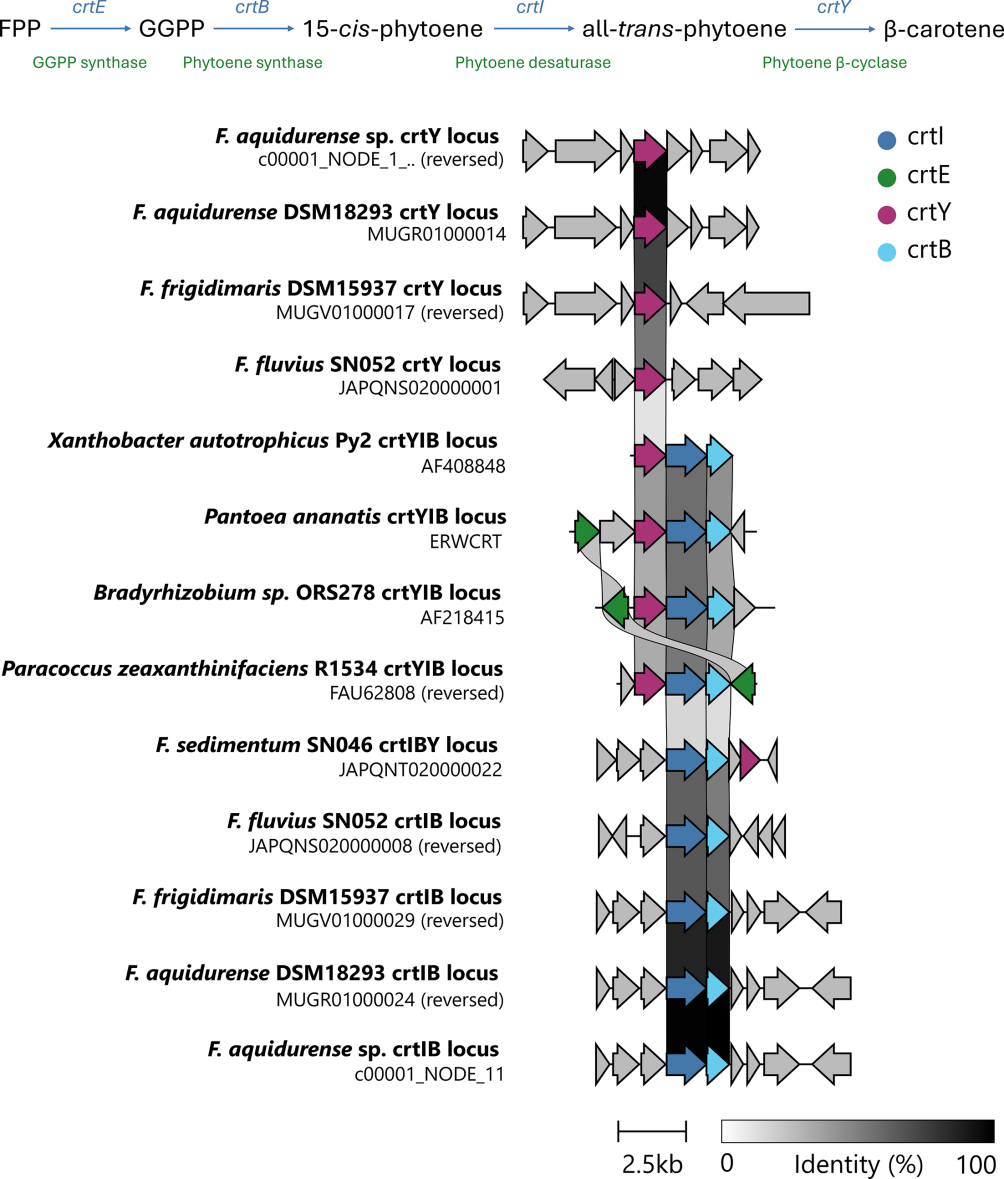
(a) β-Carotene biosynthetic gene pathway from the farnesyl pyrophosphate (FPP) precursor molecule. Gene names are highlighted in blue, while encoded protein functions are highlighted in green. GGPP, geranylgeranyl pyrophosphate (b) Organization of the β-carotene BGC, highlighting the four crt genes that are involved. The identity cut-off was <20%. The gene identities between non-crt genes are not shown. The *Flavobacterium* spp. loci containing crt genes are split by the presence of crtIB and crtY. All the comparative Pseudomonadota have a single locus that contains the crt genes. Part B was generated using clinker [40].

As the production of pigments is common for *Flavobacterium* spp*.* [1], the potentially encoded secondary metabolites were investigated using AntiSMASH v.7.0 [22]. Two main groups of pigments produced by *Flavobacterium* spp. are flexirubins and carotenoids, with only one group of compounds typically being produced per isolate; however, some isolates do produce both [1]. Analysis of the AntiSMASH output showed potential for flexirubin production, with 32 of the 35 genes within the single biosynthetic gene cluster (BGC) showing greater than 80% nucleotide identity to the flexirubin BGC from *Flavobacterium johnsoniae* [23]. In addition, the four essential β-ketoacyl synthase genes within this BGC showed between 89% and 94% nucleotide identity (100% coverage). Genes involved in β-carotene production were also present within the sequenced isolate (Table 1), one of each phytoene synthase (*crtB*), phytoene desaturase (*crtI*) and lycopene β-cyclase (*crtY*) genes (Fig. 1) [24]. A pair of *crtB and crtI* genes was adjacently located on contig 11, with the remaining *crtY* gene being on contig 1. In many other bacterial species, the majority from the phylum Pseudomonadota and also *Flavobacterium sedimentum* SUN046, all three genes are co-localized within a single BGC [25,27]. However, this is not the case for the *F. aquidurense* isolate reported here, and several other *Flavobacterium* spp. (Fig. 1 and Table 1) [5,8, 26]. Carotenoid biosynthesis is under-explored in *Flavobacterium* spp., as the only study previously characterizing *Flavobacterium* sp. strain R1534 carotenoid biosynthesis; the strain was later re-classified as *Paracoccus zeaxanthinifaciens* ATCC 21588 [25,28]. The genes identified herein were compared for similarity to the *crt* genes found in * F. aquidurense, F. frigidimaris, F. sedimentum, F. fluvius* and the reclassified *P. zeaxanthinifaciens* (Table 1). This comparative analysis highlights the high levels of nucleotide similarity within the BGCs of *Flavobacterium* spp., but low similarity to * P. zeaxanthinifaciens,* with the exception being no significant nucleotide identity to the *crtY* gene in *F. sedimentum*, which was reported as a rare *crtY_cd_* variant [26]. AntiSMASH analysis also predicted the presence of genes whose encoded proteins could synthesize a novel β-lactone-type compound. β-lactones are strained heterocyclic four-membered rings, which can be used as intermediates during total synthesis, and β-lactone-containing natural products are increasingly being found to have bioactivity as enzyme inhibitors [29]. This prediction is based on the presence of two genes, one encoding an hydroxymethylglutaryl-CoA lyase(HMGL)-like protein and one encoding an AMP-binding protein, within the same BGC [29,30]. The putative BGC was also compared to the MIBiG 4.0 database of previously characterized BGCs, but no significant matches were found [22,31].

**Table 1.** β-Carotene biosynthetic genes compared between *Flavobacterium* spp. isolates and *P. zeaxanthinifaciens*

Gene name	Gene function	Genome location	Locus tag	Amino acid sequence identity (coverage)				
				*F. aquidurense* DSM18293	*F. frigidimaris* DSM15937	*F. fluvius* SUN052	*F. sedimentum* SUN046	*P. zeaxanthinifaciens* ATCC 21588
*crtB*	Phytoene synthase	Contig 11:36067–36906	ACGI8V_20220	100% (100%)	92.1% (100%)	68.46% (100%)	57.97% (99%)	23.9% (92%)
*crtI*	Phytoene desaturase	Contig 11:34597–36063	ACGI8V_20215	99.2% (100%)	88.52% (100%)	76.08% (99%)	65.42% (98%)	25.67% (98%)
*crtY*	Lycopene β-cyclase	Contig 1:107776–108981	ACGI8V_00530	97.8% (100%)	80.2% (98%)	59.54% (97%)	n/a	19.5% (95%)

As some *Flavobacterium* spp. are considered pathogenic, the resistance gene profile was explored using the Comprehensive Antibiotic Resistance Database (CARD [32]) and ResFinder [33]. We identified the β-lactamase resistance gene *bla_JOHN-1_* (77.8% amino acid sequence identity, 81.4% nucleotide sequence identity), originally found in *F. johnsoniae* [34] and confirmed the presence of the six residues required for zinc coordination in subclass-B1 metallo-β-lactamases (His116, His118, Asp120, His196, Cys221 and His263) (consensus class B1 numbering [35]). The draft genome sequence was also screened for the presence of mobile genetic elements (MGEs) using MobileElementFinder [36]. There were two low identity matches to IS*3* family MGEs, IS*Fnu6* [37], 70.2% nucleotide identity across 35.6% of the sequence, and IS*Enfa3* [38], 72.3% nucleotide identity over 26.1% sequence coverage. However, these putative novel insertion sequences were not associated with *bla_JOHN-1_* and were present on different contigs.

This work indicates a widespread geographic distribution following this isolation in north-west England in addition to previously being isolated in central Germany [3]. The initial mischaracterization of our strain as *F. frigidimaris* based on 16S rRNA sequence comparison indicates that using the *gyrB* sequence for initial *Flavobacterium* species assignment is a preferable taxonomic indicator, which agrees with previous findings [2]. The discovery of a *bla_JOHN-1_* analogue within *F. aquidurense* indicates that this carbapenemase is more widespread than its initial discovery within *F. johnsoniae* [34] and suggests that *Flavobacterium* spp. could generally represent a β-lactamase reservoir. Finally, the pigment and β-lactone biosynthetic gene pathways described above show that *F. aquidurense* could be a useful source of natural products with multiple industrial applications [29,39].

## Abbreviations

ANI: average nucleotide identity
BGC: biosynthetic gene cluster
BHI: brain-heart infusion
CARD: Comprehensive Antibiotic Resistance Database
FPP: farnesyl pyrophosphate
GGPP: geranylgeranyl pyrophosphate
HMGL: hydroxymethylglutaryl-CoA lyase
MGE: mobile genetic elements
TE: tris ethylenediaminetetraacetic acid
